# Discovery of Potent c-MET Inhibitors with New Scaffold Having Different Quinazoline, Pyridine and Tetrahydro-Pyridothienopyrimidine Headgroups

**DOI:** 10.3390/molecules21050612

**Published:** 2016-05-11

**Authors:** Yingnan Jiang, Ke Zhang, Suyu Gao, Guihua Wang, Jian Huang, Jinhui Wang, Lixia Chen

**Affiliations:** School of Traditional Chinese Materia Medica, Shenyang Pharmaceutical University, Shenyang 110016, China; j1074736705@163.com (Y.J.); xjzk1984@163.com (K.Z.); gsy19900922@163.com (S.G.); redticky@126.com (G.W.); 13504051049@163.com (J.H.)

**Keywords:** quinazoline, pyridine, tetrahydro-pyridothienopyrimidine, MET inhibitor, cancer therapy

## Abstract

Cellular mesenchymal-epithelial transition factor (c-MET) is closely linked to human malignancies, which makes it an important target for treatment of cancer. In this study, a series of 3-methoxy-*N*-phenylbenzamide derivatives, *N*-(3-(*tert*-butyl)-1-phenyl-1*H*-pyrazol-5-yl) benzamide derivatives and *N*^1^-(3-fluoro-4-methoxyphenyl)-*N*^3^-(4-fluorophenyl) malonamide derivatives were designed and synthesized, some of them were identified as c-MET inhibitors. Among these compounds with new scaffolds having different quinazoline, pyridine and tetrahydro-pyridothienopyrimidine head groups, compound **11c**, **11i**, **13b**, **13h** exhibited both potent inhibitory activities against c-MET and high anticancer activity against tested cancer cell lines *in vitro*. In addition, kinase selectivity assay further demonstrated that both **13b** and **13h** are potent and selective c-MET inhibitors. Molecular docking supported that they bound well to c-MET and VEGFR2, which demonstrates that they are potential c-MET RTK inhibitors for cancer therapy.

## 1. Introduction

**Figure 1:**
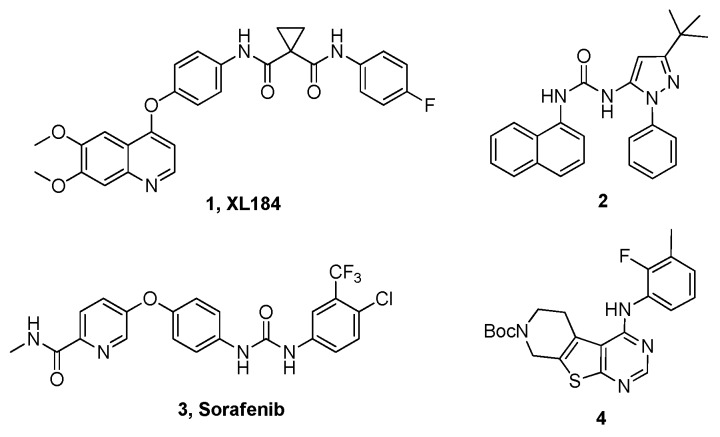
Representative c-MET inhibitor (**1**, **XL184**) and Multi-kinase inhibitor (**2**–**4**).

Tyrosine kinase is an enzyme that transfers a phosphate group from ATP to a protein, and it functions as an “on” or “off” switch in many cellular functions. They become potent oncogene that has the potential to cause cancer, when they are often mutated or expressed at high levels [1]. Several receptor tyrosine kinases (RTKs) inhibitors have been found to have effective anti-tumor activity and some of them have been approved or are in clinical trials. Recent FDA approved drugs Sorafenib (Nexavar) [2] is such example of multi-targeted agents (Figure 1).

Cellular mesenchymal-epithelial transition factor (c-MET), the hepatocyte growth factor receptor (HGFR), belongs to a subfamily of RTK that are composed of an extracellular a chain and a membrane-spanning b chain connected through a disulfide bond [3,4,5]. Binding of Hepatocyte Growth Factor/Scatter Factor (HGF/SF) to c-MET induces phosphorylation of tyrosine residues on c-MET and activates its downstream signaling pathway [6,7], which is associated with cell proliferation, migration, invasion and survival and is essential for normal embryonic development and wound healing [8,9]. However, it is reported that the c-MET/HGF axis is also involved in the development of various human malignancies. Aberrant or mutated expression of c-MET/HGF axis has been observed in a number of malignancies such as breast, gastric, bladder and lung cancers, which is closely linked to tumorigenesis and metastasis [10,11,12]. Moreover, it is reported that dysregulation of c-MET also correlated with a poor prognosis in clinical studies [13]. Therefore, c-MET shows high potential as a therapeutic target for human cancer.

Well-known agents for targeting the c-MET/HGF axis include anti-HGF antibodies, anti-c-MET antibodies, and c-MET tyrosine kinase inhibitors (TKIs). Among them, c-MET TKIs are the most attractive means to target c-MET pathway, because they are thought to be effective against both ligand-dependent and ligand-independent activation of c-MET [14]. In recent years, a number of c-MET inhibitors have been reported or have entered clinical trials, and many of them are c-MET/VEGFR-2 (vascular endothelial growth factor receptor 2) dual inhibitors (see Figure 1) [15,16,17]. For example, Cabozantinib (**XL184**, BMS-907351) is a potent c-MET inhibitor with IC_50_ of 1.3 nM and also inhibits VEGFR2, Ret, Kit, Flt-1/3/4, Tie2, and AXL in cell-free assays, respectively. Compounds **2** and **4** represent potential lead compounds with c-MET inhibitory effect in high throughput screening [18,19]. There are two classes of c-MET inhibitors based on their chemical structures or binding modes, but they both have some shortcomings [20,21]. Class I inhibitors bind in a U-shaped conformation to the ATP-binding site at the entrance of a kinase pocket, wrap around Met1211 and bind to a hinge-block, and thus specifically inhibiting MET kinases, such as Crizotinib. Class II inhibitors bind to a region of MET that extends from the ATP binding site to Ile1145 near the C-C-spiral block, such as Cabozantinib. Studies suggested that class II inhibitors maybe more effective than class I inhibitors, but they exhibited off-target effects of other protein kinases, and clinical use of class II inhibitors have shown that these agents cause serious toxic effects in many organs [22,23,24,25]. Therefore, potent c-MET inhibitors with improved selectivity and minimal side effects should be developed.

In this study, we disclose our efforts towards the design and synthesis of new c-MET inhibitors. To further understand the structure–activity relationship (SAR) of this novel series of compounds, different quinazoline, pyridine and tetrahydro-pyridothienopyrimidine fragments were investigated. A series of 3-methoxy-*N*-phenylbenzamide derivatives, *N*-(3-(*tert*-butyl)-1-phenyl-1*H*-pyrazol-5-yl) benzamide derivatives and *N*^1^-(3-fluoro-4-methoxyphenyl)-*N*^3^-(4-fluorophenyl) malonamide derivatives were synthesized, and their inhibitory activities against c-MET and three different cancer cell lines were evaluated. We were interested to see if such modifications were possible within our new classes of molecules compared to the parent compounds.

## 2. Results and Discussion

### 2.1. Chemistry

**Scheme 1:**
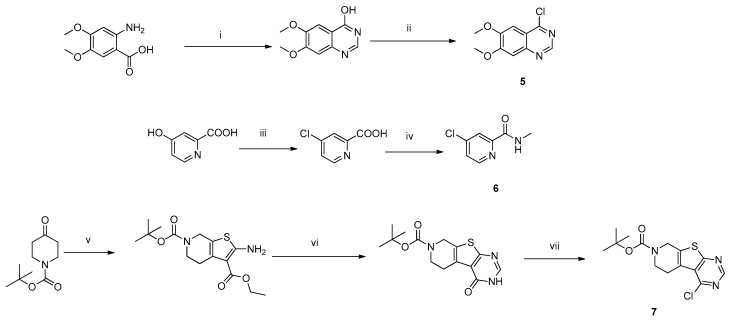
The synthesis procedure of the three different headgroups. *Reagents and conditions*: (i) Formamidine acetate, CH_3_OCH_2_CH_2_OH, 125 °C, 16 h; (ii) THF, SOCl_2_, reflux, 3 h; (iii) DMF, SOCl_2_, 120 °C, 6 h; (iv) THF, EDCI, CH_3_NH_2_, rt., 3 h; (v) S, NCCH_2_CO_2_Et, EtOH, Et_3_N, rt, 16 h; (vi) Formamidine acetate, DMF, 120 °C, 16 h; (vii) POCl_3_, DIPEA, toluene, 80 °C, 12 h.

The chemistry described in Scheme 1 shows the synthetic route chosen to obtain quinazolines (Compound **5**), pyridines (Compound **6**) and tetrahydro-pyridothienopyrimidines (Compound **7**) fragments. The reaction of 2-amino-4,5-dimethoxybenzoic acid with formamidine acetate afforded intermediate 6,7-dimethoxyquinazolin-4-ol, which upon reaction with SOCl_2_ afforded compound **5**. The synthetic method of compound **7** is similar to the above process [26,27].

**Scheme 2:**
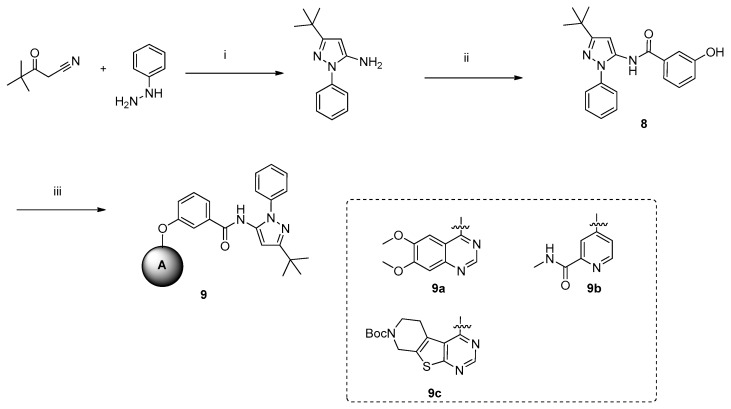
The synthesis procedure of Compound **9a**–**c**. *Reagents and conditions*: (i) EtOH, reflux, 16 h; (ii) THF, 3-hydroxybenzoic acid, ClCOCOCl, 0 °C, 3 h; (iii) **5**, **6** or **7**, DMF, K_2_CO_3_, 80 °C, 24 h.

The chemistry described in Scheme 2 shows compound libraries could be made simply by using the reaction protocol with *N*-(3-(*tert*-butyl)-1-phenyl-1*H*-pyrazol-5-yl)-3-hydroxybenzamide (Compound **8**) and different head groups. A cyclization reaction with nitrile and hydrazine was carried to afford 3-(*tert*-butyl)-1-phenyl-1*H*-pyrazol-5-amine. Compound **8** was synthesized by a conventional peptide synthesis method using ClCOCOCl at 0 °C [28].

**Scheme 3:**
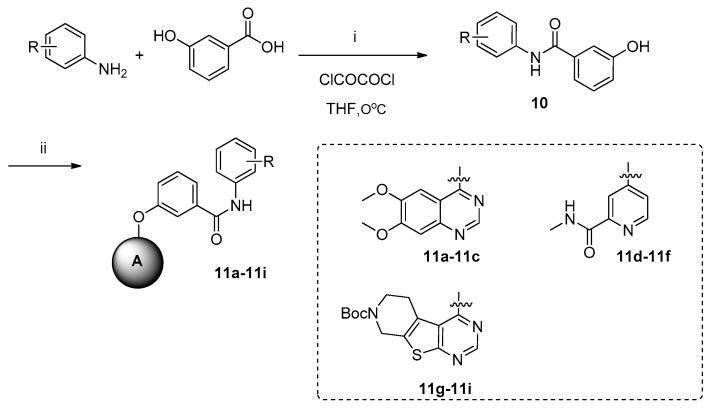
The synthesis procedure of Compound **11a**–**i**. *Reagents and conditions*: (i) THF, ClCOCOCl, 0 °C, 3 h; (ii) **5,** **6** or **7**, DMF, K_2_CO_3_, 80 °C, 24 h.

Inspired by the structure of the lead compound **4**, we have also designed a series of *N*-phenylbenzamide derivatives. The synthetic route is shown in Scheme 3. Compounds **10** were synthesized by a conventional peptide synthesis method with aniline and benzoic acid using ClCOCOCl at 0 °C. Then, a typical Williamson ether synthesis was carried on to afford compound **11a**–**i** [29].

**Scheme 4:**
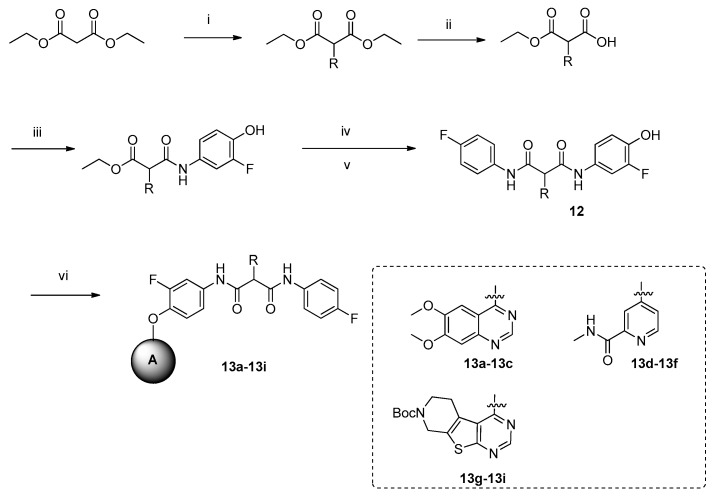
The synthesis procedure of Compound **13a**–**i**. *Reagents and conditions*: (i) EtONa, CH_3_I, 0 °C, 5 h, or 1,2-dibromoethane or 1,3-dibromopropane, Bu_4_NBr, K_2_CO_3_, DMF, rt., 12 h; (ii) KOH, EtOH, 0 °C, 5 h; (iii) 4-amino-2-fluorophenol, IBCF, NMM, THF, −15 °C, 5 h; (iv) KOH, EtOH, 0 °C, 5 h; (v) 4-fluoroaniline, IBCF, NMM, THF, −15 °C, 3 h; (vi) **5,** **6** or **7**, DMF, K_2_CO_3_, 80 °C, 24 h.

We also combined different head groups to the side chain of lead compound **1**, **XL184**. The synthetic route is shown in Scheme 4. After multi-step protection and de-protection reaction, a series ethyl hydrogen malonate derivative was obtained. Then, Compounds **12** were synthesized by a conventional peptide synthesis method using isobutyl chloroformate and 4-Methylmorpholine. After that, a typical Williamson ether synthesis was carried on to afford compound **13a**–**i** [30].

### 2.2. Biology

#### 2.2.1. Kinase Inhibitory Assay

All the synthesized compounds were assayed with the enzymatic activity against c-MET. The results were summarized in Table 1, Table 2 and Table 3. Also included was the representative c-MET inhibitor (**XL184**). Among these compounds, compound **11c,** **11i,** **13b,** **13h** showed potent inhibitory activity against c-MET, with IC_50_ of 0.08 µM (**11c**), 0.05 µM (**11i**), 0.02 µM (**13b**), 0.05 µM (**13h**), which is compared to **XL184** (IC_50_ = 0.03 µM against c-MET).

#### 2.2.2. Anti-proliferation against Tumor Cells Assay

All the synthesized compounds were evaluated for their anticancer activity *in vitro* against HeLa (human cervical carcinoma), Hep-G2 (human liver cancer) and MCF-7 (human breast cancer). The results were also summarized in Table 1, Table 2 and Table 3. It is clear that compound **11c**, **11i**, **13b**, **13h** all exhibited good anticancer activity against tested cancer cell lines. Compared to **XL184** (IC_50_ = 6.6 µM against HeLa), these four compounds all showed better anti-proliferation activity against HeLa with compound **11c** (IC_50_ = 0.9 µM against HeLa) exhibiting the highest activity. Similarly, they also showed better anti-proliferation activity against Hep-G2 than **XL184** (IC_50_ = 49.1 µM), and it is obvious that compound **13h** (IC_50_ = 1.7 µM) showed the highest activity, which is far better than **XL184**. In addition, compound **11i** (IC_50_ = 1.1 µM) and **13b** (IC_50_ = 1.2 µM) had better results against MCF-7 than **XL184** (IC_50_ = 2.6 µM).

#### 2.2.3. Structure Activity Relationship Analysis

Preliminary structure–activity SAR study appeared that different head groups showed great differences in cell and enzymatic activity. Among them, quinazolines (compound **9a**, **11a**–**c**, **13a**–**c**), and tetrahydro-pyridothienopyrimidines (compound **9c**, **11g**–**i**, **13g**–**i**) head groups provided much better activity than pyridines (compound **9b**, **11d**–**f**, **13d**–**f**). For the side chain, *N*-(3-(*tert*-butyl)-1-phenyl-1*H*-pyrazol-5-yl)-3-methoxybenzamide derivatives had lowest activities while malonamide derivatives had lowest activities. For compound **13a**–**i**, different R groups provided different activity, when R group was cyclopropane (compound **13b** and **13h**), the best activity was observed.

#### 2.2.4. Enzymatic Selectivity Assay

Compound **13b** and **13h** were further assayed with enzymatic activities against VEGFR-2, c-Kit, PDGFR-b and EGFR to test their kinase selectivity [31]. The results were summarized in Table 4. Compound **13b** demonstrated extraordinary selectivity against c-Kit (215 fold), PDGFR-b (>500 fold) and EGFR (>500 fold), but it showed some inhibitory activity against VEGFR-2 (IC_50_ = 0.1 µM). Compound **13h** exhibited extraordinary selectivity against c-Kit (104 fold), PDGFR-b (144 fold) and EGFR (>200 fold), and it also showed some inhibitory activity against VEGFR-2 (IC_50_ = 0.25 µM).

### 2.3. Molecular Docking and Molecular Dynamics Simulation Study

In order to better understand the interaction between compounds and kinases, molecular docking studies on the potent compound **11c** and **11i** were performed using the Discovery Studio 3.1/CDOCKER protocol [32].

**Figure 2:**
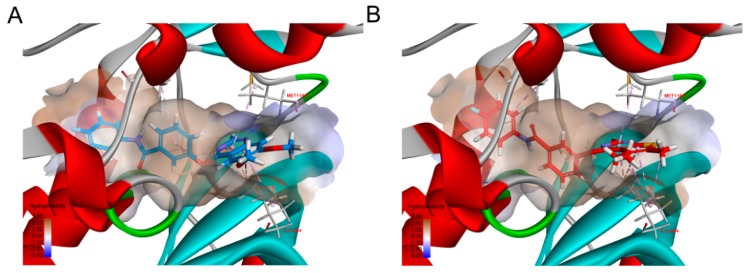
Binding poses of compound **11c** (**A**) and **11i** (**B**) with c-MET. Compound **11c** and **11i** were displayed by blue and red, respectively. The important residues and their critical interactions (dash lines) with compounds in the binding site were depicted, hydrogen bond, hydrophobic interactions were colored green and carnation, respectively.

In Figure 2, we showed that compound **11c** and **11i** could bind to c-MET kinase (PDB: 4MXC) very well. In addition, they can form hydrophobic interaction in the ATP-binding sites of c-MET and VEGFR-2. Compounds formed hydrophobic interaction with residues ILE-1084, ALA-1108, LEU-1157, MET-1160 and ALA-1221 of c-MET.

**Figure 3:**
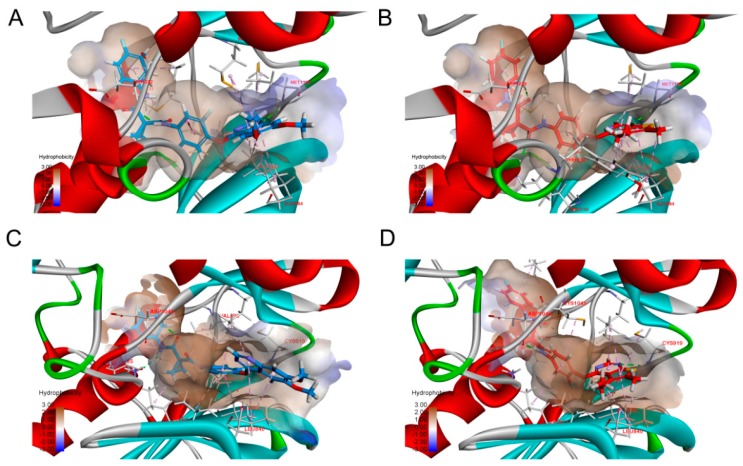
Binding poses of compound **13b** (**A**,**C**) and **13h** (**B**,**D**) with c-MET and VEGFR2. Compound **13b** and **13h** were displayed by blue and red, respectively. The important residues and their critical interactions (dash lines) with compounds in the binding site were depicted, hydrogen bond, hydrophobic interactions were colored green and carnation, respectively.

For the better active compounds **13b** and **13h**, We demonstrated in Figure 3 the compound **13b** and **13h** docking into the binding site of c-MET kinase (PDB: 4MXC) and VEGFR-2 kinase (PDB: 4ASE). For compound **13b**, there are two hydrogen bonds formed by residue MET-1160 and ASP-1222, additional, compound **13b** could form hydrophobic interactions with ILE-1084, ALA-1108, MET-1131, LEU-1157, ALA-1221 to c-MET. Compound **13b** can form three hydrogen bonds by residue GLU-885, CYS-919, and ASP-1046, there also exist hydrophobic interaction with residue LEU-840, VAL-848, ALA-866, LYS-868, LEU-889, VAL-899, VAL-916, LEU-1035 to VEGFR2. Binding energy of compound **13b** is much better than compound **11c** and **11i**, the dock interaction energy of compound **13b** with c-MET, compound **11c** and **11i** with c-MET is −70.33 kJ/mol, −44.8 kJ/mol and −47.6 kJ/mol, respectively. The nice binding model of compound **13b** and **13h** with c-MET and VEGFR-2 was consistent with kinase assay data, which indicates that compounds were potent dual c-MET/VEGFR-2 inhibitors.

To further evaluate the binding affinity between compound **13b** and c-MET/VEGFR-2. The molecular dynamics (MD) simulations were performed by using GROMACS package (version 4.5, University of Groningen, Groningen, The Netherlands), the root-mean-square deviation (RMSD) fluctuations is a principal criterion to evaluate the stability of the protein-ligand system.

**Figure 4:**
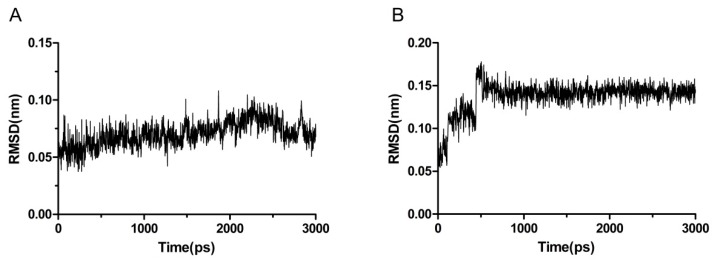
RMSD of compound **13b** with c-MET (**A**) and VEGFR-2 (**B**), respectively.

As shown in Figure 4, although the RMSD values for compound **13b** fluctuated in a narrow range around 2700 ps, they remained stable for most of the simulation in c-MET complex. RMSD of compound **13b** reach the equilibrium state after about 500 ps and kept stable among the rest of simulation in VEGFR-2 complex, indicating a stabilities of the dynamics equilibriums. In general, the maximum RMSD for each case was lower than 0.2 nm, suggesting that c-MET and VEGFR-2 complexes are reliable and the low RMSD fluctuations of system observed indicated stable binding models of compound **13b** with c-MET and VEGFR-2, respectively.

## 3. Experimental Section

### 3.1. Chemical Synthesis

All reagents were purchased from commercial sources and used without further purification. Melting points are corrected. ^1^H-NMR spectra were determined on a Bruker Avance III 400 MHz spectrometer (Bruker, Billerica, MA, USA) in CDCl_3_ or DMSO-*d*_6_ solution. *J* values were in Hz. Chemical shifts were expressed in ppm downfield from internal standard TMS. HRMS data were obtained using Bruker micro TOF-Q instrument (Bruker) or TOF-MS instrument (Bruker).

#### 3.1.1. General Procedure for the Preparation of 4-Chloro-6,7-dimethoxyquinazoline (Compound **5**)

2-amino-dimethyl aminobenzoic acid 2.02 g (10 mmol) and acetic acid formamidine 2.10 g (20 mmol) were added in 2-methoxy ethanol. The mixture was reflux in 125 °C. After evaporation of solvent, the residue was added in the 10% ammonia solution, stirring, then after filtering, the solid was washed by water to get the brown solid powder (yield 89%). 1.82 g (8.8 mmol) product was dissolved in 25 mL SOCl_2_, and 30 drops of DMF was added. After heating 7 h to reflux, the solvent was evaporated, then after filtering, the solid was washed by water to get the brown solid powder 4-chloro-6,7-dimethoxyquinazoline (yield 93%).

#### 3.1.2. General Procedure for the Preparation of {*tert*-Butyl,4-chloro-5,6-dihydropyrido(4’,3’:4,5)thieno(2,3-*d*)pyrimidine-7(8*H*)-carboxylate} (Compound **7**)

The *N*-(*tert*-Butoxycarbonyl)-4-piperidone, NCCH_2_CO_2_Et and Et_3_N was mixed at room temperature then stirring for 16 h, and then heated to 120 °C for 16 h in DMF with the formamidine acetate. Then, the intermediate was reacted with POCl_3_ and DIPEA in toluene to obtain the compound **7** (yield 89%).

#### 3.1.3. General Procedure for the Preparation of {*N*-(3-(*tert*-Butyl)-1-phenyl-1*H*-pyrazol-5-yl)-3-((6,7-dimethoxyquinazolin-4-yl)oxy)benzamide} Derivatives Compound **9a**–**c**

Pivaloylacetonitrile 4.07 g (32 mmol) and Phenylhydrazine 3.51 g (32 mmol) were added to 40 mL ethanol and the mixture was heated to reflux for 15 h. After the solvent was evaporated, [3-(*tert*-butyl)-1-phenyl-1*H*-pyrazol-5-amine] was obtained. 138.6 mg (1 mmol) hydroxybenzoic acid in 5 mL THF was added three drops of DMF and reacted with oxalyl chloride 0.5 mL in 0 °C. After 1.5 h reaction, the reaction liquid was dissolved in THF and HCl, which was dissolved in THF solution of 3-(*tert*-butyl)-1-phenyl-1*H*-pyrazol-5-amine. After 3 h, the reaction mixture was evaporated; the crude product was purified by column chromatography to obtain compound **8**. Compound **8** was further reacted with compounds **5**, **6**, **7** in the presence of K_2_CO_3_ to obtain the final products **9a**–**c**.

Compound **9a**: *N*-(3-(*tert*-butyl)-1-phenyl-1*H*-pyrazol-5-yl)-3-((6,7-dimethoxyquinazolin-4-yl)oxy)benzamide. White solid, yield 88%, m.p. 217–219 °C. ^1^H-NMR (400 MHz, DMSO) δ 10.40 (s, 1H), 8.57 (s, 1H), 7.83 (d, *J* = 7.3 Hz, 1H), 7.79 (s, 1H), 7.64 (t, *J* = 7.8 Hz, 1H), 7.60 (s, 1H), 7.57 (s, 1H), 7.54 (s, 2H), 7.52 (s, 2H), 7.44 (t, *J* = 7.8 Hz, 2H), 7.40 (s, 1H), 7.31 (t, *J* = 7.3 Hz, 1H), 6.40 (s, 1H), 4.01(s, 3H), 3.97(s, 3H), 1.31 (s, 9H). HRMS (*m*/*z*): calcd. for 524.2292 ([M + H]^+^), obsd. 524.2294.

Compound **9b**: 4-{3-((3-(*tert*-butyl)-1-phenyl-1*H*-pyrazol-5-yl)carbamoylphenoxy}-*N*-methylpicolinamide: yellow solid, yield 71%. ^1^H-NMR (400 MHz, CDCl_3_) δ 8.78 (d, 5.6 Hz, 1H), 8.35–7.95 (m, 2H), 7.94–7.67 (m, 5H), 7.62–7.42 (m, 2H), 7.39–7.05 (m, 2H), 6.74 (s, 1H), 2.85 (s, 3H), 1.29 (s, 9H). ^13^C-NMR (100 MHz, CDCl_3_ ) δ 169.26, 167.39, 166.32, 158.34, 153.42, 151.87, 148.57, 146.98, 139.89, 135.40, 129.43, 128.96, 128.93, 125.18, 123.62, 118.50, 113.11, 112.47, 95.64, 34.41, 28.32, 26.37.HRMS (*m*/*z*): calcd. for 470.2192 ([M + H]^+^), obsd. 470.2181.

Compound **9c**: *tert*-butyl 4-{3-((3-(*tert*-butyl)-1-phenyl-1*H*-pyrazol-5-yl)carbamoyl)phenoxy}-5,6-dihydropyrido(4’,3’:4,5)thieno(2,3-*d*)pyrimidine-7(8*H*)-carboxylate, yellow solid, yield 71%. ^1^H-NMR (400 MHz, CDCl_3_) δ 8.01–7.67 (m, 5H), 7.67–7.42 (m, 2H), 7.43–7.02 (m, 2H), 4.65–4.55 (m, 2H), 3.70–3.40 (m, 2H), 3.20–3.15 (m, 2H), 1.42 (s, 9H), 1.29 (s, 9H). ^13^C-NMR (100 MHz, CDCl_3_) δ 170.28, 169.27, 162.31, 157.47, 154.66, 153.42, 151.87, 148.98, 139.89, 138.69, 129.19, 128.96, 128.93, 126.61, 126.27, 125.18, 122.57, 122.30, 119.22, 117.01, 95.64, 81.20, 43.81, 42.71, 34.41, 28.32, 23.62. HRMS (*m*/*z*): calcd. for 625.2597 ([M + H]^+^), obsd. 625.2611.

#### 3.1.4. General Procedure for the Preparation of 3-Methoxy-*N*-phenylbenzamide Derivatives Compound **11a**–**i**

138.6 mg (1 mmol) hydroxybenzoic acid in 5 mL THF was added three drops of DMF and reacted with oxalyl chloride 0.5 mL in 0 °C. After 1.5 h reaction, the reaction liquid was dissolved in THF and HCl, which was dissolved in THF solution of aniline derivatives. After 3 h, the reaction mixture was evaporated; the crude product was purified by column chromatography to obtain compounds **10**. Compounds **10** were further reacted with compounds **5**, **6**, **7** in the presence of K_2_CO_3_ to obtain the final products **11a**–**i**.

Compound **11a**: *N*-(3,4-dimethoxyphenyl)-3-((6,7-dimethoxyquinazolin-4-yl)oxy)benzamide. Yellow solid, yield 86%, m.p. 220–222 °C. ^1^H-NMR (400 MHz, CDCl_3_) δ 8.62 (s, 1H), 7.90 (s, 1H), 7.81 (d, *J* = 7.4 Hz, 2H), 7.60 (t, *J* = 8.2 Hz, 1H), 7.53 (s, 1H), 7.47 (t, 2H), 7.33 (s, 1H), 6.99 (dd, *J* = 8.6, 2.3 Hz, 1H), 6.84 (d, *J* = 8.6 Hz, 1H), 4.07 (d, *J* = 2.8 Hz, 6H), 3.90 (d, *J* = 5.2 Hz, 3H), 3.87 (s, 3H). ^13^C-NMR (100 MHz, CDCl_3_) δ 166.79, 165.05, 154.75, 153.84, 153.57, 149.95, 148.67, 148.21, 138.69, 132.68, 129.19, 126.27, 122.57, 122.29, 118.41, 114.98, 113.43, 111.44, 108.20, 107.06, 56.83.HRMS (*m*/*z*): calcd. for 462.1660 ([M + H]^+^), obsd.462.1665. calcd. for 484.11478 ([M + Na]^+^), obsd.484.1490.

Compound **11b**: *N*-(4-chlorophenyl)-3-((6,7-dimethoxyquinazolin-4-yl)oxy)benzamide. Faint yellow solid, yield 89%, m.p. 203–205 °C. ^1^H-NMR (400 MHz, DMSO) δ 10.42 (s, 1H), 8.58 (s, 1H), 7.94 (s, 1H), 7.92 (s, 1H), 7.83 (s, 1H), 7.80 (s, 1H), 7.67 (t, *J* = 7.8 Hz, 1H), 7.60 (s, 1H), 7.58 (s, 1H), 7.41 (t, 3H), 4.00 (d, *J* = 5.4 Hz, 6H). ^13^C-NMR (100 MHz, DMSO) δ 165.19, 162.11, 155.85, 154.74, 153.57, 147.81, 137.54, 136.45, 130.37, 129.25, 128.67, 127.37, 122.88, 122.46, 121.79, 114.79, 110.88, 107.91, 57.24.HRMS (*m*/*z*): calcd. for 420.1354 ([M + H]^+^), obsd.436.1059. calcd. for 458. 0878 ([M + Na]^+^), obsd.458.0883.

Compound **11c**: 3-((6,7-dimethoxyquinazolin-4-yl)oxy)-*N*-(4-fluorophenyl)benzamide. Yellow solid, yield 86%, m.p. 178–180 °C. ^1^H-NMR (400 MHz, DMSO) δ 10.35 (s, 1H), 8.58 (s, 1H), 7.94 (s, 2H), 7.92 (d, *J* = 2.3 Hz, 2H), 7.79 (q, 1H), 7.67 (t, *J* = 7.8 Hz, 1H), 7.62–7.56 (m, 2H), 7.42 (s, 1H), 7.20 (t, *J* = 7.2 Hz, 2H), 4.00 (d, *J* = 5.2 Hz, 6H). ^13^C-NMR (100 MHz, DMSO) δ 167.33, 166.05, 162.07, 159.55, 154.65, 153.84, 153.12, 148.21, 138.69, 134.01, 129.03, 125.77, 122.62, 122.03, 115.11, 111.14, 108.30, 55.43.HRMS (*m*/*z*): calcd. for 420.1354 ([M + H]^+^), obsd.420.1356. calcd. for 442.1174 ([M + Na]^+^), obsd.442.1185.

Compound **11d**: 4-{3-((3,4-dimethoxyphenyl)carbamoyl)phenoxy}-*N*-methylpicolinamide: yellow solid, yield 82%, ^1^H-NMR (400 MHz, CDCl_3_ ) δ 8.78 (d, *J* = 5.6 Hz, 1H), 8.22–7.89 (m, 2H), 7.67–7.37 (m, 2H), 7.34 (s, 1H), 7.27–7.04 (m, 3H), 6.91 (d, *J* = 15.4 Hz, 1H), 6.48 (s, 1H), 3.83 (s, 3H), 3.76 (s, 3H), 2.85 (s, 3H). ^13^C-NMR (100 MHz, CDCl_3_) δ 168.12, 167.12, 164.25, 157.14, 149.75, 148.47, 147.57, 146.98, 135.32, 132.18, 129.23, 123.62, 118.50, 117.10, 113.43, 113.11, 112.47, 107.06, 56.83, 26.37. HRMS (*m*/*z*): calcd. for 407.1481 ([M + H]^+^), obsd. 407.1499.

Compound **11e**: 4-{3-((4-chlorophenyl)carbamoyl)phenoxy}-*N*-methylpicolinamide: yellow solid, yield 68%. ^1^H-NMR (400 MHz, CDCl_3_) δ 8.62 (d, *J* = 5.6 Hz, 1H), 8.01–7.78 (m, 2H), 7.81–7.62 (m, 2H), 7.65–7.42 (m, 2H), 7.48–7.29 (m, 3H), 7.16 (m, 1H), 6.52 (s, 1H), 2.85 (s, 3H). ^13^C-NMR (100 MHz, CDCl_3_) δ 171.77, 169.22, 165.15, 159.14, 149.17, 147.28, 136.46, 135.21, 131.37, 129.44, 127.45, 124.57, 121.78, 118.60, 114.31, 113.17, 24.77. HRMS (*m*/*z*): calcd. for 382.0958 ([M + H]^+^), obsd. 382.0961.

Compound **11f**: 4-{3-((4-fluorophenyl)carbamoyl)phenoxy}-*N*-methylpicolinamide: yellow solid, yield 82%, ^1^H-NMR (400 MHz, CDCl_3_ ) δ 8.74 (d, *J* = 5.6 Hz, 1H), 8.02–7.76 (m, 2H), 7.72–7.46 (m, 4H), 7.34 (m, 1H), 7.21–7.01 (m, 3H), 6.50 (s, 1H), 2.85 (s, 3H). ^13^C-NMR (100 MHz, CDCl_3_) δ 169.39, 168.32, 167.05, 163.07, 159.55, 158.34, 148.57, 146.98, 135.40, 134.03, 132.01, 128.43, 123.62, 122.66, 121.58, 118.51, 115.10, 113.90, 112.17, 110.47, 28.37. HRMS (*m*/*z*): calcd. for 407.1481 ([M + H]^+^), obsd. 407.1499.

Compound **11g**: *tert*-butyl 4-{3-((3,4-dimethoxyphenyl)carbamoyl)phenoxy}-5,6-dihydropyrido(4’,3’:4,5)thieno(2,3-*d*)pyrimidine-7(8*H*)-carboxylate: yellow solid, yield 71%, m.p. 103–105 °C. ^1^H-NMR (400 MHz, CDCl_3_ ) δ 8.92 (s, 1H), 8.50 (s, 1H), 7.65–7.40 (m, 2H), 7.34 (m, 1H), 7.29–7.07 (m, 3H), 6.91 (m, 1H), 4.70 (s, 2H), 3.81 (s, 3H), 3.78 (s, 3H), 3.59–3.43 (m, 2H), 3.17 (m, 2H), 1.42 (s, 9H). ^13^C-NMR (100 MHz, CDCl_3_) δ 170.28, 166.05, 162.31, 157.47, 154.66, 149.95, 148.98, 148.67, 138.69, 132.68, 129.19, 126.61, 126.27, 122.57, 122.30, 119.22, 118.40, 117.01, 113.43, 107.06, 81.20, 56.83, 43.81, 42.71, 28.33, 23.62. HRMS (*m*/*z*): calcd. for 563.1964 ([M + H]^+^), obsd. 563.1921.

Compound **11h**: *tert*-butyl 4-{3-((4-chlorophenyl)carbamoyl)phenoxy}-5,6-dihydropyrido(4’,3’:4,5)thieno(2,3-*d*)pyrimidine-7(8*H*)-carboxylate: yellow solid, yield 74%, ^1^H-NMR (400 MHz, CDCl_3_) δ 8.50 (s, 1H), 7.81–7.67 (m, 2H), 7.61–7.42 (m, 2H), 7.42–7.16 (m, 4H), 4.65 (s, 2H), 3.67–3.40 (m, 2H), 3.17 (m, 2H), 1.42 (s, 9H). ^13^C-NMR (100 MHz, CDCl_3_ ) δ 173.11, 168.12, 164.45, 159.26, 155.78, 149.12, 139.57, 136.46, 130.37, 129.26, 128.01, 127.74, 126.77, 123.78, 122.86, 121.41, 118.12, 116.21, 79.31, 44.25, 43.78, 27.32, 24.42. HRMS (*m*/*z*): calcd. for 537.1363 ([M + H]^+^), obsd. 537.1378.

Compound **11i**: *tert*-butyl 4-{3-((4-fluorophenyl)carbamoyl)phenoxy}-5,6-dihydropyrido(4’,3’:4,5)thieno(2,3-*d*)pyrimidine-7(8*H*)-carboxylate: yellow solid, yield 77%, ^1^H-NMR (400 MHz, CDCl_3_) δ 8.50 (s, 1H), 7.73–7.43 (m, 4H), 7.34–7.15 (m, 4H), 4.65 (s, 2H), 3.65–3.42 (m, 2H), 3.18 (m, 2H), 1.42 (s, 9H). ^13^C-NMR (100 MHz, CDCl_3_ ) δ 171.01, 167.25, 163.42, 162.01, 159.45, 158.27, 155.84, 147.88, 137.32, 135.05, 134.00, 130.12, 127.42, 125.77, 123.66, 122.57, 121.70, 119.62, 118.71, 116.10, 114.93, 82.18, 44.28, 43.54, 27.43, 24.55. HRMS (*m*/*z*): calcd. for 521.1659 ([M + H]^+^), obsd. 521.1711.

#### 3.1.5. General Procedure for the Preparation of *N*^1^-(3-Fluoro-4-hydroxyphenyl)-*N*^3^-(4-fluorophenyl)malonamide Derivatives Compounds **12**

Diethyl malonate 2.53 mL (17 mmol), iodomethane or 1,2-dibromoethane or 1,3-dibromoethane (22 mmol), K_2_CO_3_ 5.7 g (42 mmol) and tetrabutylammonium bromide 0.27 g (0.08 mmol) were added in DMF. The mixture is stirred for 16 h at room temperature. After evaporation of solvent, the residue was extracted with ethyl acetate 100 mL three times, and the organic layer was washed with water. After the concentration of the solvent, colorless oil diethyl cyclopropane-1,1-dicarboxylatederivatives were obtained (yield 89%).

Diethyl cyclopropane-1,1-dicarboxylatederivatives (1.0 eq.) were dissolved in EtOH. KOH (1.0 eq.) in ethanol solution was added and stirred for 5 h. After the completion of the reaction, the intermediate was dissolved in THF solution at −15 °C. 4-Methylmorpholine (1.2 eq.), isobutyl chloroformate (1.0 eq.) and 4-amino-2-fluorophenol (1.0 eq.) were added. The crude product was purified by column chromatography, and the above process was repeated. The final product compounds **12** were obtained.

#### 3.1.6. General Procedure for the Preparation of *N*^1^-(3-Fluoro-4-methoxyphenyl)-*N*^3^-(4-fluorophenyl)malonamide Derivatives Compound **13a**–**i**

Compounds **12** were further reacted with compounds **5**, **6**, **7** in the presence of K_2_CO_3_ to obtain the final products **13a**–**i** as mentioned above.

Compound **13a**: *N*^1^-{4-((6,7-dimethoxyquinazolin-4-yl)oxy)-3-fluorophenyl}-*N*^3^-(4-fluorophenyl)-2,2-dimethylmalonamide. White solid, yield 85%, m.p. 210–212 °C. ^1^H-NMR (400 MHz, CDCl_3_) δ 8.84 (s, 1H), 8.51 (s, 1H), 8.22 (s, 1H), 7.70 (m, 1H), 7.48 (s, 1H), 7.44–7.23 (m, 2H), 7.23–7.15 (m, 1H), 6.97 (t, *J* = 8.6 Hz, 1H), 3.99 (d, *J* = 4.3 Hz, 1H), 1.62 (s, 1H). ^13^C-NMR (100 MHz, CDCl_3_) δ 172.19, 171.92, 165.66, 162.17, 159.64, 156.11, 155.44, 153.27, 152.81, 150.33, 149.47, 136.40, 136.30, 136.20, 136.07, 133.02, 124.10, 122.59, 115.91, 110.17, 109.39, 109.15, 106.81, 100.93, 56.36, 50.76, 24.18. HRMS (*m*/*z*): calcd. for 523.1788 ([M + H]^+^), obsd.535.1791.

Compound **13b**: *N*-{4-[(6,7-dimethoxyquinazolin-4-yl)oxy]-3-fluorophenyl}-*N*-(4-fluorophenyl)cyclopropane-1,1-dicarboxamide. Yellow solid, yield 86%, m.p. 180–182 °C. ^1^H-NMR (400 MHz, CDCl_3_) δ 9.66 (s, 1H), 8.52 (s, 1H), 8.47 (s, 1H), 7.67 (d, *J* = 11.1 Hz, 1H), 7.48 (s, 1H), 7.38 (dd, *J* = 9.0, 4.8 Hz, 1H), 7.20 (d, *J* = 5.5 Hz, 1H), 6.98 (t, *J* = 8.6 Hz, 1H), 3.99 (d, *J* = 4.5 Hz, 3H), 1.66 (t, *J* = 6.1 Hz, 1H), 1.58–1.51 (m, 1H). ^13^C-NMR (100 MHz, CDCl_3_) δ 169.43, 168.43, 164.68, 156.04, 152.69, 150.36, 149.45, 136.42, 136.03, 124.07, 122.95, 122.87, 116.10, 115.96, 115.73, 110.20, 109.63, 109.40, 106.78, 100.96, 56.37, 31.93, 29.70, 29.29, 22.69, 17.61, 14.12. HRMS (*m*/*z*): calcd. for 521.1631 ([M + H]^+^), obsd.521.1634.

Compound **13c**: *N*-{4-((6,7-dimethoxyquinazolin-4-yl)oxy)-3-fluorophenyl}-*N*-(4-fluorophenyl)cyclobutane-1,1-dicarboxamide. White solid, yield 80%, m.p. 250–252 °C. ^1^H-NMR (400 MHz, CDCl_3_) δ 8.58 (s, 1H), 8.53 (d, *J* = 7.2 Hz, 1H), 8.16 (d, *J* = 7.5 Hz, 1H), 7.80 (dd, *J* = 11.9, 2.1 Hz, 1H), 7.57–7.48 (m, 1H), 7.35–7.30 (m, 1H), 7.28 (d, *J* = 3.4 Hz, 1H), 7.03 (t, *J* = 8.5 Hz, 1H), 4.06 (d, *J* = 4.5 Hz, 1H), 2.06–1.90 (m, 1H), 1.28–1.23 (m, 1H). ^13^C-NMR (101 MHz, CDCl_3_) δ 170.54, 170.40, 164.67, 160.91, 158.47, 156.02, 155.52, 153.05, 152.69, 150.34, 149.44, 136.64, 136.12, 133.38, 124.14, 122.02, 121.94, 115.88, 115.65, 110.16, 109.09, 108.86, 106.78, 100.93, 56.36, 56.02, 29.76, 15.67. HRMS (*m*/*z*): calcd. for 535.1788 ([M + H]^+^), obsd.535.1793.

Compound **13d**: *N*^1^-(4-fluorophenyl)-2,2-dimethyl-*N*^3^-{4-((2-(methylcarbamoyl)pyridin-4-yl)oxy]phenyl}malonamide. Faint yellow solid, yield 82%, m.p. 113–115 °C. ^1^H-NMR (400 MHz, CDCl_3_) δ 8.72 (s, 1H), 8.54 (s, 1H), 8.37 (d, *J* = 5.6 Hz, 1H), 8.02 (d, *J* = 4.0 Hz, 1H), 7.66 (d, *J* = 1.9 Hz, 1H), 7.60–7.50 (m, 3H), 7.09–6.99 (m, 4H), 6.94 (dd, *J* = 5.6, 2.5 Hz, 1H), 2.99 (d, *J* = 5.1 Hz, 3H), 1.70 (s, 6H). ^13^C-NMR (100 MHz, CDCl_3_) δ 171.71, 166.36, 164.46, 160.93, 158.50, 152.26, 150.23, 149.67, 135.10, 133.32, 122.47, 122.39, 122.32, 121.43, 115.81, 115.59, 114.10, 110.12, 50.69, 29.70, 26.15, 25.36, 24.24. HRMS (*m*/*z*): calcd. for 451.1776 ([M + H]^+^), obsd.451.1779. Calcd. for 473.1596 ([M + Na]^+^), obsd.473.1602.

Compound **13e**: *N*-(4-fluorophenyl)-*N*-{4-((2-(methylcarbamoyl)pyridin-4-yl)oxy)phenyl}cyclopropane-1,1-dicarboxamide. White solid, yield 82%, m.p. 138–140 °C. ^1^H-NMR (400 MHz, CDCl_3_) δ 9.46 (s, 2H), 8.31 (d, *J* = 5.6 Hz, 1H), 7.99 (d, *J* = 4.9 Hz, 1H), 7.54–7.50 (m, 2H), 7.49 (s, 1H), 7.43–7.36 (m, 1H), 6.99 (s, 1H), 6.96 (d, *J* = 2.6 Hz, 1H), 6.94 (s, 1H), 6.91 (4, 1H), 4.03 (q, 1H), 2.88 (d, *J* = 5.1 Hz, 3H), 1.19 (t, 2H), 1.13 (d, *J* = 6.1 Hz, 2H). ^13^C-NMR (100 MHz, CDCl_3_) δ 169.65, 169.34, 166.39, 164.64, 152.09, 150.24, 149.81, 135.18, 133.43, 123.21, 122.91, 121.42, 115.72, 115.50, 114.39, 109.72, 64.43, 60.43, 28.89, 26.18, 25.35, 21.05, 17.91, 14.19. HRMS (*m*/*z*): calcd. for 624.2087 ([M + H]^+^), obsd.449.1620. calcd. for 471.1439 ([M + Na]^+^), obsd.471.1464.

Compound **13f**: *N*-(4-fluorophenyl)-*N*-{4-((2-(methylcarbamoyl)pyridin-4-yl)oxy)phenyl}cyclobutane-1,1-dicarboxamide. White solid, yield 77%, m.p. 192–194 °C. ^1^H-NMR (400 MHz, CDCl_3_) δ 8.29 (s, 1H), 8.27 (d, *J* = 5.1 Hz, 1H), 8.14 (s, 1H), 7.94 (d, *J* = 4.5 Hz, 1H), 7.60–7.56 (m, 1H), 7.54 (s, 1H), 7.49–7.38 (m, 1H), 6.96 (dd, *J* = 8.6, 4.5 Hz, 1H), 6.92 (s, 1H), 6.86 (dd, *J* = 5.5, 2.5 Hz, 1H), 2.92 (d, *J* = 5.1 Hz, 1H), 2.68 (t, *J* = 7.9 Hz, 1H), 2.01–1.88 (m, 1H). ^13^C-NMR (100 MHz, CDCl_3_) δ 170.49, 170.45, 166.31, 164.53, 152.28, 150.10, 149.71, 135.33, 121.96, 121.88, 121.81, 121.43, 115.80, 115.57, 114.09, 110.09, 64.42, 55.91, 29.78, 26.14, 25.35, 15.68. HRMS (*m*/*z*): calcd. for 463.1776 ([M + H]^+^), obsd.463.1779. calcd. for 485.1596 ([M + Na]^+^), obsd.485.1602.

Compound **13g**: *tert*-butyl-4-{2-fluoro-4-(3-((4-fluorophenyl)amino)-2,2-dimethyl-3-oxopropanamido)phenoxy}-5,6-dihydropyrido(4’,3’:4,5)thieno(2,3-*d*)pyrimidine-7(8*H*)-carboxylate. Faint yellow solid, yield 75%, m.p. 126–128 °C. ^1^H-NMR (400 MHz, CDCl_3_) δ 8.92 (s, 1H), 8.48 (s, 1H), 8.24 (s, 1H), 7.75 (dd, 1H), 7.53–7.45 (m, 2H), 7.25–7.18 (m, 2H), 7.05 (t, *J* = 8.6 Hz, 2H), 4.74 (s, 2H), 3.80 (t, *J* = 5.4 Hz, 2H), 3.18 (s, 2H), 1.69 (s, 6H), 1.51 (s, 9H). ^13^C-NMR (100 MHz, CDCl_3_) δ 171.94, 171.13, 169.06, 162.68, 159.13, 158.67, 154.55, 152.49, 136.38, 135.74, 135.61, 132.99, 124.02, 122.59, 122.51, 118.09, 115.93, 115.81, 115.71, 109.35, 109.11, 80.57, 50.74, 31.93, 29.70, 29.66, 29.37, 28.43, 24.20, 22.70, 19.55, 14.12. HRMS (*m*/*z*): calcd. for 624.2087 ([M + H]^+^), obsd.624.2091. Calcd. for 646.1906 ([M + Na]^+^), obsd.646.1913.

Compound **13h**: *tert*-butyl-4-{2-fluoro-4-(1-((4-fluorophenyl)carbamoyl)cyclopropanecarboxamido)phenoxy}-5,6-dihydropyrido(4’,3’:4,5)thieno(2,3-*d*)pyrimidine-7(8*H*)-carboxylate. White solid, yield 82%, m.p. 171–173 °C. ^1^H-NMR (400 MHz, CDCl_3_) δ 9.76 (s, 1H), 8.52 (s, 1H), 8.41 (s, 1H), 7.65–7.34 (m, 3H), 7.22–7.12 (m, 2H), 6.97 (t, *J* = 8.6 Hz, 2H), 4.66 (s, 2H), 3.72 (t, *J* = 5.6 Hz, 2H), 3.11 (s, 2H), 1.54 (dd, *J* = 7.7, 4.9 Hz, 2H), 1.43 (s, 9H), 1.18 (t, *J* = 7.1 Hz, 4H). ^13^C-NMR (100 MHz, CDCl_3_) δ 169.56, 169.03, 168.49, 162.68, 161.13, 158.69, 155.43, 154.58, 152.97, 152.49, 136.55, 136.45, 135.69, 135.56, 132.87, 123.95, 123.02, 118.21, 116.19, 115.93, 115.81, 109.70, 109.47, 80.70, 60.54, 29.80, 29.34, 28.53, 21.16, 17.65, 14.25. HRMS (*m*/*z*): calcd. for 622.1930 ([M+H]^+^), obsd.622.1930. Calcd. for 644.1750 ([M + Na]^+^), obsd.644.1793.

Compound **13i**: *tert*-butyl-4-{2-fluoro-4-(1-((4-fluorophenyl)carbamoyl)cyclobutanecarboxamido)phenoxy}-5,6-dihydropyrido(4’,3’:4,5)thieno(2,3-*d*)pyrimidine-7(8*H*)-carboxylate. Yellow solid, yield 83%, m.p. 101–103 °C. ^1^H-NMR (400 MHz, CDCl_3_) δ 8.48 (s, 1H), 8.36 (s, 1H), 7.96 (s, 1H), 7.78 (dd, *J* = 11.9, 2.1 Hz, 1H), 7.56–7.41 (m, 2H), 7.27–7.18 (m, 2H), 7.04 (t, *J* = 8.6 Hz, 2H), 4.74 (s, 2H), 3.80 (t, *J* = 5.4 Hz, 2H), 3.18 (s, 2H), 2.85–2.65 (m, 4H), 2.05–1.99 (m, 2H), 1.51 (s, 9H). ^13^C-NMR (100 MHz, CDCl_3_) δ 170.48, 170.16, 169.07, 162.68, 160.91, 158.51, 155.46, 154.54, 152.99, 152.47, 136.60, 135.79, 135.55, 134.32, 133.24, 131.70, 124.05, 121.99, 121.91, 118.09, 115.91, 115.68, 115.47, 109.04, 108.81, 80.56, 61.84, 55.94, 31.93, 29.75, 29.70, 28.43, 15.68. HRMS (*m*/*z*): calcd. for 636.2087 ([M + H]^+^), obsd.636.2068. Calcd. for 658.1906 ([M + Na]^+^), obsd.658.1912.

### 3.2. Cell Proliferative Assay

The anti-proliferative activities of synthesized compounds against HeLa, Hep-G2 and MCF-7 cells were evaluated by MTT assay [10]. HeLa cells were grown to log phase in RPMI 1640 medium supplemented with 10% fetal bovine serum. Hep-G2 and MCF-7 cells were grown to log phase in DMEM medium supplemented with 10% fetal bovine serum. After diluting to 5 × 10^5^ cells∙L^−1^ with the complete medium, 100 µL of the obtained cell suspension was added to each well of 96-well culture plates. The subsequent incubation was permitted at 37 °C, 5% CO_2_ atmosphere for 48 h before the anti-proliferative assessments. Tested samples at pre-set concentrations were added to 6 wells. Before the anti-proliferative assessments, 20 µL of PBS containing 2.5 mg∙mL^−1^ of MTT was added to each well. Four h later, 100 µL extraction solution (DMSO) was added to each well and the optical density was measured at a wavelength of 570 nm on an ELISA microplate reader.

### 3.3. In Vitro Kinase Assay

*In vitro* kinase inhibitory ability was determined using HTScan MET Kinase Assay Kit, HTScan VEGF Receptor 2 Kinase Assay Kit, HTScan c-Kit Kinase Assay Kit, HTScan PDGF Receptor β Kinase Assay Kit and HTScan EGFR Kinase Assay Kit(purchased from Cell Signaling Technology, Inc. Danvers, MA, USA) by colorimetric ELISA assay according to the manufacturer’s instructions. Briefly, reaction cocktail containing recombinant human MET kinase or other kinases were incubated with various concentrations of tested compounds or DMSO (0.1%) for 5 min at room temperature, and then ATP/substrate peptide cocktail was added to the pre-incubated reaction cocktail. After incubation at room temperature for 30 min, the reaction was stopped and transferred to a 96-wellstreptavidin-coated plate, and incubated for 1 h at room temperature. Primary antibody (phosphorylated tyrosine monoclonal antibody (pTyr-100), 1:1000 in PBS/T with 1% bovine serum albumin (BSA)) was added into per well until the wells were washed thrice with PBS/T. After incubated at room temperature for 1 h, phosphorylation of the substrate was monitored with HRP-labeled anti-mouse IgG antibody (1:500 in PBS/T with 1% BSA), followed by a chromogenic reaction. Finally, the kinase assay was detected at 450 nm with microplate reader. The reaction processed with only DMSO (0.1%) served as a vehicle control. The results were expressed as percent kinase activity of the vehicle control, and IC_50_ was defined as the compound concentration that resulted in 50% inhibition of enzyme activity. The kinase assay was performed thrice independently.

### 3.4. Molecular Dockingand Dynamics Simulation

Molecular docking of compounds into the three dimensional X-ray structure of c-MET (PDB code: 4MXC) and VEGFR-2 (PDB code: 4ASE) was carried out using the Discovery Studio (version3.1) as implemented through the graphical user interface Discovery Studio CDOCKER protocol [32].

The three-dimensional structure of all compounds was constructed using ChemBio 3D Ultra 11.0 software (Chemical Structure Drawing Standard; Cambridge Soft corporation, Waltham, MA, USA), then it was energetically minimized by using MMFF94 with 5000iterations and minimum RMS gradient of 0.10. The crystal structures of VEGFR-2 kinase were retrieved from the RCSB Protein Data Bank (http://www.rcsb.org/pdb/home). All bound waters and ligands were eliminated from the protein and the polar hydrogen was added. The whole protein structure was defined as are acceptor and the site sphere was selected based on ATP binding site of 4ASE or 4MXC. Compounds were placed during the molecular docking procedure. Types of interactions of the docked protein with ligand were analyzed after the end of molecular docking.

To further confirm the binding model of compound **13b** with c-MET and VEGFR-2, respectively, MD simulations was performed using the GROMACS package (version 4.5, University of Groningen) [33]. By using the AMBER99 force field and general AMBER force field (gaff), receptor and ligands were charged, respectively [34]. The simulations study were carried out under periodic boundary conditions (PBC) with minimum distance (0.1 nm) between the kinase and the edge of the box. The complex of compound **13b** with protein were solvated in a cubic box of TIP3P water molecules and neutralized by adding Na^+^ and Cl^−^ to mimic physiological NaCl concentration of 0.15 M. Prior to MD simulations, energy minimization were performed to remove bad contacts, 100-ps NVT and 100-ps NPT ensembles with protein and ligand position restraints were carried out to equilibrate each system. Finally, 3-ns MD production simulations were carried out for each system with a 2-fs time step at constant pressure (1 atm) and temperature (300 K).

## 4. Conclusions

In this study, a series of 3-methoxy-*N*-phenylbenzamide derivatives, *N*-(3-(*tert*-butyl)-1-phenyl-1*H*-pyrazol-5-yl) benzamide derivatives and *N*^1^-(3-fluoro-4-methoxyphenyl)-*N*^3^-(4-fluorophenyl) malonamide derivatives were synthesized, and some of them were identified as potent and selective c-MET inhibitors. Compound **13b** showed the most potent inhibitory activity against c-MET with IC_50_ of 0.02 µM, and it exhibited good anticancer activity against tested cancer cell lines. In addition, compound **11c**, **11i**, **13h** also demonstrated potent c-MET inhibitory activity and excellent anticancer activity *in vitro*. Importantly, compound **13h** showed the highest anti-proliferation activity against Hep-G2 with IC_50_ of 1.7 µM, which is better than the c-MET inhibitor **XL184**, suggesting that compound **13h** may have great potential for liver cancer therapy. Kinase selectivity assay suggested that compound **13b** and **13h** also showed a little inhibitory activity against VEGFR-2. Moreover, molecular docking results disclosed binding modes of these potent inhibitors and c-MET. Therefore, compound **11c**, **11i**, **13b**, **13h** are potent c-MET inhibitors and could be potential anticancer agents.

## Figures and Tables

**Table 1:** Enzymatic and cellular results for compound **9a**–**c**. 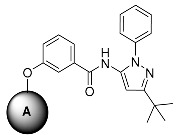

Compound	A	Enzymatic Inhibition (IC_50_, µM) ^a,b^	Proliferative Inhibition (IC_50_, µM) ^b^		
		c-MET	HeLa	Hep-G2	MCF-7
**9a**	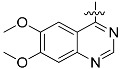	8.4 ± 0.6	28.7 ± 1.1	38.5 ± 1.3	46.1 ± 0.8
**9b**	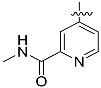	>10	>100	>100	59.7 ± 1.3
**9c**	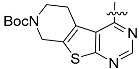	>10	>100	>100	66.7 ± 1.5

^a^ Prior to the initiation of the kinase reaction with ATP (10 µM), compounds and enzyme were incubated for 30 min. ^b^ IC_50_ values obtained with viability assays. Each value is representative of three independent determinations.

**Table 2:** Enzymatic and cellular results for compound **11a**–**c**. 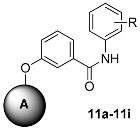

Compound	A	R	Enzymatic Inhibition (IC_50_, µM) ^a,b^	Proliferative Inhibition (IC_50_, µM) ^b^		
			c-MET	HeLa	Hep-G2	MCF-7
**11a**	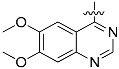	3,4-diOMe	0.52 ± 0.05	14.1 ± 0.16	>100	3.4 ± 0.21
**11b**		4-Cl	0.12 ± 0.03	1.8 ± 0.06	9.2 ± 0.17	7.8 ± 0.14
**11c**		4-F	0.08 ± 0.04	0.9 ± 0.12	10.1 ± 0.20	5.2 ± 0.36
**11d**	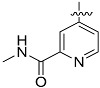	3,4-diOMe	>10	>100	>100	71.2 ± 0.34
**11e**		4-Cl	7.4 ± 0.07	26.4 ± 0.20	>100	34.5 ± 0.36
**11f**		4-F	5.3 ± 0.05	27.8 ± 0.34	>100	42.1 ± 0.27
**11g**	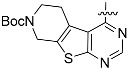	3,4-diOMe	1.4 ± 0.10	4.4 ± 0.26	>100	17.3 ± 0.38
**11h**		4-Cl	0.25 ± 0.08	10.7 ± 0.18	61.9 ± 0.23	22.9 ± 0.22
**11i**		4-F	0.05 ± 0.02	1.5 ± 0.11	23.2 ± 0.36	1.1 ± 0.31
**XL184**			0.03 ± 0.02	2.6 ± 0.07	49.1 ± 0.25	6.6 ± 0.25

^a^ Prior to the initiation of the kinase reaction with ATP (10 µM), compounds and enzyme were incubated for 30 min. ^b^ IC_50_ values obtained with viability assays. Each value is representative of three independent determinations.

**Table 3:** Enzymatic and cellular results for compound **13a**–**c.** 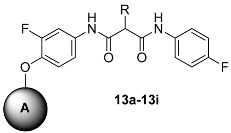

Compound	A	R	Enzymatic Inhibition (IC_50_, µM) ^a,b^	Proliferative Inhibition (IC_50_, µM) ^b^		
			c-MET	HeLa	Hep-G2	MCF-7
**13a**	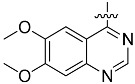	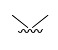	7.1 ± 0.18	>100	>100	71.8 ± 0.31
**13b**		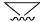	0.02 ± 0.09	4.7 ± 0.11	12.8 ± 0.28	1.2 ± 0.21
**13c**		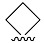	1.2 ± 0.06	50.1 ± 0.25	49.1 ± 0.30	>100
**13d**	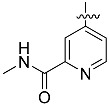	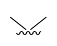	>10	>100	>100	91.2 ± 0.26
**13e**		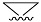	>10	66.7 ± 0.36	>100	>100
**13f**		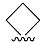	>10	87.8 ± 0.13	>100	>100
**13g**	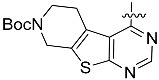	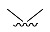	9.4 ± 0.6	>100	>100	27.7 ± 0.35
**13h**		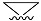	0.05 ± 0.01	4.6 ± 0.03	1.7 ± 0.04	2.8 ± 0.23
**13i**		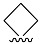	0.25 ± 0.08	4.2 ± 0.31	43.2 ± 0.26	21.7 ± 0.16
**XL184**			0.03 ± 0.02	6.6 ± 0.11	49.1 ± 0.13	2.6 ± 0.03

^a^ Prior to the initiation of the kinase reaction with ATP (10 µM), compounds and enzyme were incubated for 30 min. ^b^ IC_50_ values obtained with viability assays. Each value is representative of three independent determinations.

**Table 4:** Kinase selectivity of compounds **13b** and **13h**.

Compound	Enzyme, IC_50_, µM ^a,b^			
	VEGFR-2	c-Kit	PDGFR-b	EGFR
**13b**	0.1 ± 0.02	4.3 ± 0.11	>10	>10
**13h**	0.25 ± 0.02	5.2 ± 0.09	7.2 ± 0.10	>10

^a^ Prior to the initiation of the kinase reaction with ATP (10 µM), compounds and enzyme were incubated for 30 min. ^b^ IC_50_ values obtained with viability assays. Each value is representative of three independent determinations.
